# Storage Effects on the Physicochemical Properties, Phytochemical Composition, and Sugars in Red-Fleshed Cultivars, ‘Rubycot’ Plumcot, and ‘Queen Garnet’ Plum

**DOI:** 10.3390/molecules29194641

**Published:** 2024-09-29

**Authors:** Gethmini Kavindya Kodagoda, Hung Trieu Hong, Tim J. O’Hare, Bruce Topp, Yasmina Sultanbawa, Michael Erich Netzel

**Affiliations:** 1 Queensland Alliance for Agriculture and Food Innovation, The University of Queensland, Coopers Plains, QLD 4108, Australia; k.kodagoda@uq.net.au (G.K.K.); y.sultanbawa@uq.edu.au (Y.S.); 2 Biosecurity Queensland, Department of Agriculture and Fisheries, Health and Food Science Precinct, Coopers Plains, QLD 4108, Australia; 3 School of Agriculture and Food Sustainability, The University of Queensland, St. Lucia, QLD 4072, Australia; h.trieu@uq.edu.au; 4 Queensland Alliance for Agriculture and Food Innovation, The University of Queensland, Gatton, QLD 4343, Australia; t.ohare@uq.edu.au; 5 Queensland Alliance for Agriculture and Food Innovation, The University of Queensland, Nambour, QLD 4560, Australia; b.topp@uq.edu.au; 6 ARC Industrial Transformation Training Centre for Uniquely Australian Foods, Queensland Alliance for Agriculture and Food Innovation, The University of Queensland, Indooroopilly, QLD 4068, Australia

**Keywords:** plumcot, plum, phytochemicals, anthocyanins, carotenoids, domestic storage, stability, nutritional quality

## Abstract

Domestic storage conditions can have a significant impact on the composition of phytochemicals and sugars in stone fruits. This study aimed to evaluate the effect of two domestic storage temperatures (4 and 23 °C) on the physicochemical properties, phytochemical composition, and sugars of ‘Rubycot’ (RC) plumcot, a novel stone fruit variety, and ‘Queen Garnet’ (QG) plum. Initially, RC had a lower total anthocyanin concentration (TAC) than QG, but TAC in RC increased significantly (*p* < 0.05) during storage, peaking at +95% after 7 days at 23 °C, while QG reached +60% after 14 days. At 4 °C, TAC increased for both varieties (RC +30%, QG +27%). RC had a higher initial total phenolic content (TPC), which also increased for both fruits. QG had a significantly higher initial total quercetin concentration (TQC), increasing by 40% (*p* < 0.05) at 23 °C. The initial total carotenoid concentration in QG was higher than that in RC, but after 10 days at 23 °C, RC had a higher carotenoid concentration than QG. Both varieties showed similar sugar profiles, with QG starting higher but decreasing over time at both storage temperatures. Results from this study showed that ambient storage significantly increases total anthocyanins, total quercetins, and TPC in RC and QG. However, it is important to evaluate the textural and sensory properties of stored RC and QG in terms of consumer acceptability of the stored fruits.

## 1. Introduction

Diet plays an important role in maintaining human health, as an unbalanced diet will not provide all the necessary macro- and micronutrients needed to maintain basic functions in the human body. Many epidemiological studies have shown that there is an inverse relationship between fruit and vegetable consumption and the occurrence of chronic diseases such as cardiovascular diseases, cancers, heart, and neurodegenerative diseases [1,2,3,4,5,6,7]. Fruits and vegetables provide not only macro- and micronutrients but also a vast variety of phytochemicals with potential preventative effects [8]. Phytochemicals are naturally occurring, non-nutritive, secondary metabolites of plants that have a range of biochemical and physiological effects. Phytochemicals can be classified as carotenoids, polyphenols, alkaloids, nitrogen-containing compounds, and organosulfur-containing compounds [9].

Plant breeding has developed new varieties of stone fruit with desirable traits such as high yield, improved internal and external quality characteristics such as colour, flavour, taste, disease, and pathogen resistance, as well as tolerance against harsh environmental conditions. Plumcots are a novel variety of stone fruit, an interspecific hybrid between Japanese plums (*Prunus salicina* L.) and apricots (*P. armeniaca*) [10]. In 2009, ‘Rubycot’ (RC) plumcot was released in Queensland, Australia, by the Department of Employment, Economic Development and Innovation (DEEDI) in a stone fruit breeding programme [11]. RC is a hybrid between the ‘Satsuma’ Japanese blood plum and an unnamed apricot variety. RC is a red-fleshed plumcot that has favourable marketable potential as a novel stone fruit due to its availability between the main seasons for apricots and plums.

Plums are highly perishable and easily deteriorate during harvesting, transportation, and storage [12,13,14]. They are usually harvested at early maturity stages before ripening and, for the extension of their shelf life, stored at 0–5 °C [15]. Normally, when consumers purchase these stone fruits from the market, they tend to store them for a few days in their households before consumption. Plums can be stored either refrigerated (4 °C) or at ambient temperature (23 °C) until they are fully ripe (soft and aromatic). During storage, based on the storage temperature and time, physicochemical and phytochemical changes may occur in plums/plumcots, altering their palatability as they continue to ripen. Though RC has been released commercially and grown in different regions of Australia, to the best of our knowledge, no studies have been conducted regarding its physicochemical properties, phytochemical composition, or postharvest storage characteristics. In this study, variations in physiological properties, phytochemicals, and sugar composition in RC plumcot stored at two domestic storage conditions (4 and 23 °C) were investigated and compared with a well-established and commercially important plum, ‘Queen Garnet’ (QG).

## 2. Results and Discussion

### 2.1. Physicochemical Properties

The consumer acceptability of plums depends on parameters such as appearance, texture, and flavour. Physicochemical parameters such as total soluble solids, titratable acidity, and colour have been widely used as indicators to evaluate the fruit quality of plums [16]. In this study, total soluble solids (TSS), titratable acidity (TA), and peel colour of QG and RC were analysed on different storage days at the two selected domestic storage temperatures (Table 1). In QG at both storage temperatures, TSS increased (trend only, *p* > 0.05) during storage. In RC, there was a non-significant (*p* > 0.05) increase in TSS at 23 °C and 4 °C, and TSS was reduced by 3% (trend only, *p* > 0.05). The initial TA was higher in RC compared to QG (2.18 vs. 1.27% of mallic acid). Interestingly, TA significantly (*p* < 0.05) decreased in both fruits at 23 °C, −23% in RC, and −43% in QG at the last storage day. Plum flavour is mainly determined by the sweetness or sourness of the plums, and the balance between acids and sugars highly influences the overall flavour of plums. Plums with a high TSS:TA ratio and TSS above 14% usually have better consumer acceptability [17]. RC and QG had TSS values above 14% at both storage temperatures. However, to evaluate the acceptability of stored RC and QG, detailed consumer sensory tests are needed.

**Figure 1:**
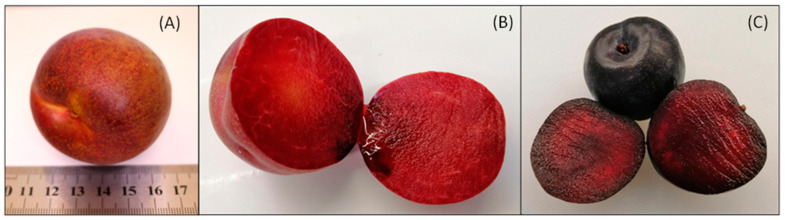
(**A**,**B**) RC plumcot, (**C**) QG plum.

Both chroma and hue angles of RC were higher than those of QG, and these values clearly distinguish the dark red colour of RC from the dark purple colour of QG (Figure 1). Similar observations were reported by other authors regarding hue angle and chroma being higher in yellow plum cultivars when compared with dark purple cultivars [18,19,20]. In RC, hue angles were non-significantly decreased at 23 °C, while at 4 °C there was a non-significant (*p* > 0.05) increase at day 10. In contrast, the hue angles of QG reduced significantly (*p* < 0.05) at both storage temperatures. Furthermore, the chroma values of QG were significantly (*p* < 0.05) reduced at 23 °C, from 9.18 at day 0 to 3.36 at day 14. However, a non-significant (*p* > 0.05) increase in chroma was observed at 4 °C, similar to the results reported for QG harvested in 2018/2019 [21]. Similar patterns of increase and decrease in chroma were observed in RC.

### 2.2. Total Phenolic Content (TPC)

**Figure 2:**
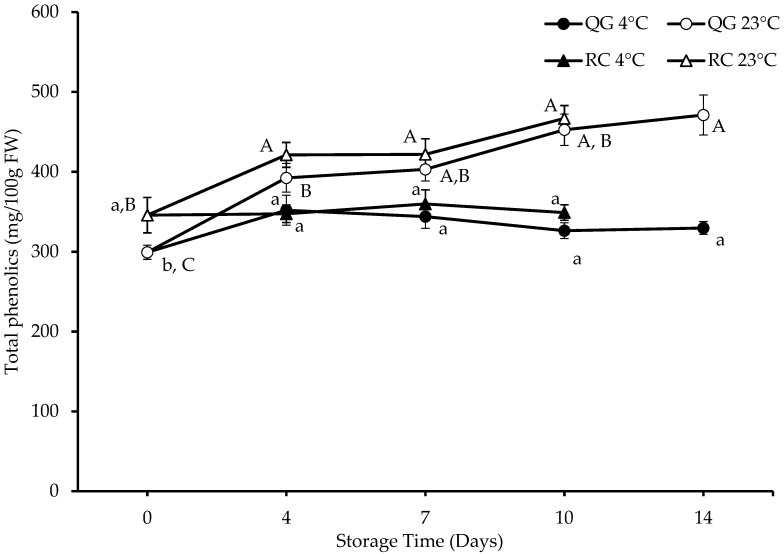
TPC (as gallic acid equivalents) in RC and QG. Data are presented as mean ± SE (*n* = 4–6). Different uppercase and lowercase letters indicate significant (*p* < 0.05) differences between storage days for each storage temperature for each fruit type.

The TPC of RC on day 0 was higher than that of QG (346 vs. 299 mg GAE/100 g FW) (Figure 2). The TPC of QG significantly (*p* < 0.05) increased at ambient (23 °C) storage, and the increase was highest on day 14 (+57% vs. day 0). At 4 °C, the highest TPC was observed after 4 days of storage (+18% vs. day 0). Though the initial TPC of RC was higher than that of QG, at the end of the storage trial at 23 °C, the TPC of QG was higher than that of RC. For each cultivar, there was a significant difference (*p* < 0.05) in TPC between the two storage temperatures for each storage day.

Polyphenols are the largest and most abundant group of phytochemicals found in human diets [9]. Polyphenols are subdivided into flavonoids and non-flavonoids, which are further subdivided into different flavonoid groups and phenolic acids based on their chemical structure. These phenolic compounds can have multiple biological activities and potential health benefits due to their anti-inflammatory, anti-diabetic, and anti-carcinogenic properties [22]. Plums have relatively high levels of phenolic compounds, especially the red and purple varieties [23].

The reported TPC in plums varies from 55 to 375 mg GAE/100 g [24,25,26,27]. The TPC of RC and QG in the present study is in the upper range of the reported content (346 and 299 mg GAE/100 g FW, respectively). However, the TPC of plums can vary greatly due to cultivar. Rupasinghe, et al. [28] reported the TPC of 20 different plum cultivars grown at the same location under similar pre-harvest conditions, ranging from 86 to 413 mg GAE/100 g FW. This suggests that the genetic variability between the different cultivars may affect the biosynthesis of phenolic compounds in the plums. Furthermore, the environment and agronomic factors can also have a significant impact on the TPC in plums. A negative correlation between the air temperature and the concentration of phenolics has been reported in different plum cultivars [29,30]. A higher humidity during the harvest time can cause less abiotic stress, which can affect transpiration and photosynthetic activity, resulting in an increased TPC in the plum [31]. Other factors such as maturity, postharvest treatment (e.g., transport, storage), and analytical methods [27,32] are also critical for the TPC in plums. Finally, it should be mentioned that the TPC method is a spectrophotometric assay and therefore relatively unspecific. The assay measures (poly)phenolic compounds and non-phenolic compounds such as ascorbic acid, which can also react with the Folin–Ciocalteu reagent. The TPC assay is, in principle, another antioxidant assay determining the reducing capacity of a compound or sample [33,34]. Therefore, TPC results should not be overrated and interpreted with some caution.

### 2.3. Anthocyanins

**Figure 3:**
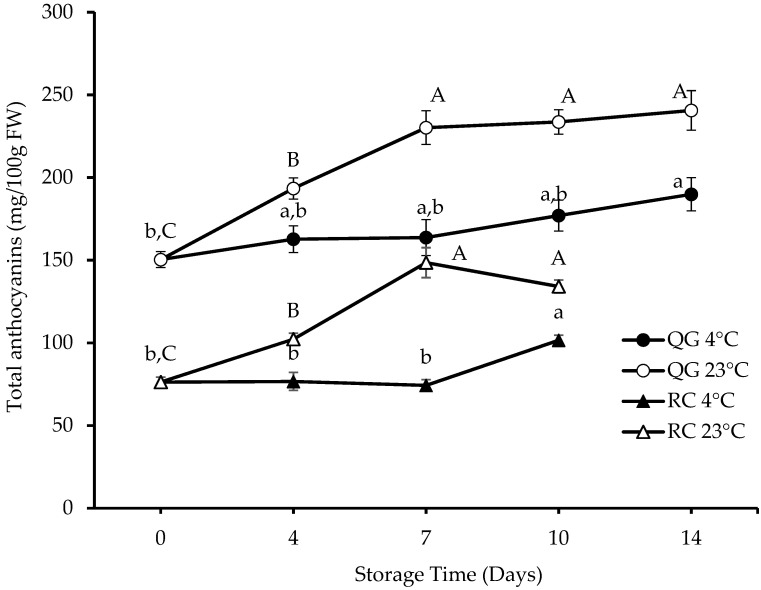
Total anthocyanin concentration of RC and QG during storage. Data are presented as mean ± SE (*n* = 4–6). Different uppercase and lowercase letters indicate significant (*p* < 0.05) differences between storage days for each storage temperature.

When compared with RC, QG had a higher anthocyanin concentration (150.4 vs. 76.3 mg/100 g FW on day 0) (Figure 3). However, this was not surprising, since QG is described in the literature as a high anthocyanin plum, having a dark, anthocyanin-rich flesh [21,35]. Cyanidin-3-glucoside and cyanidin-3-rutinoside are the main anthocyanins in QG and RC, with cyanidin-3-glucoside as the predominant anthocyanin in QG (57% of TAC) and cyanidin-3-rutinoside as the predominant anthocyanin in RC (45% of TAC). Two other anthocyanin pigments, peonidin-3-glucoside and peonidin-3-rutinoside, could also be found in RC (18% of TAC) (Table 2). Peonidin-3-glucoside, peonidin-3-rutinoside, and other peonidin derivatives were also identified in some European plum cultivars [36,37,38]. Although carotenoids are the main pigments in apricots, some apricot varieties also contain anthocyanins, which give them a red blush on the yellow or orange skin. Cyanidin-3-glucoside, cyanidin-3-rutinoside, and peonidin-3-rutinoside were identified in these red-blushed apricots [39,40,41]. Recent findings suggest that the anthocyanin production in these red-blushed apricots depends on sunlight and is directly regulated by the R2R_3_ MYB TF *PaMYB10* gene [42].

At 23 °C storage, the TAC of QG increased up to day 14 to a final concentration of 241 mg/100 g FW, whereas the TAC of RC reached a maximum of 149 mg/100 g FW at day 7. At 4 °C storage, the TAC in both QG and RC continued to increase until the last day of storage. However, the increase was considerably lower when compared with the increase at 23 °C (RC: 33% vs. 95%; QG: 27% vs. 60%). For each cultivar, there was a significant difference (*p* < 0.05) in TAC between the two storage temperatures for each storage day. Previous studies have reported that the reduction in the hue angle of dark-coloured plums during storage is associated with the production of anthocyanins [19,43]. Similar reductions in the hue angle were observed in QG, but not in RC (Table 1). To the best of our knowledge, this is the first time that such changes in the anthocyanin content of plumcots have been reported.

### 2.4. Quercetins

**Figure 4:**
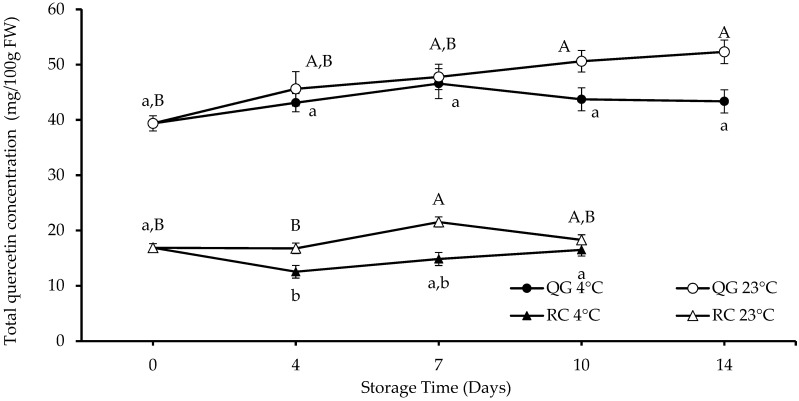
Total quercetin concentrations (calculated as quercetin-3-glucoside equivalents) in RC and QG during storage. Data are presented as mean ± SE (*n* = 4–6). Different uppercase and lowercase letters indicate significant (*p* < 0.05) differences between storage days for each storage temperature.

Quercetin glycosides and quercetin are the most common flavonols in plums. Seven different quercetin derivatives were tentatively identified in QG and RC as shown in Table 3. QG had a 2.5 times higher initial total quercetin concentration (TQC) than RC (Figure 4). There was a significant increase (40%) in TQC in QG stored at 23 °C, and also an increase (16%; *p* > 0.05, trend only) at 4 °C. However, no significant differences or a “clear” trend (increase or decrease) were observed during the storage of RC at both temperatures. In RC, there were significant differences (*p* < 0.05) in TQC between the two storage temperatures, except on day 10. For QG, a significant difference (*p* < 0.05) in TQC was observed only on days 10 and 14.

**Figure 5:**
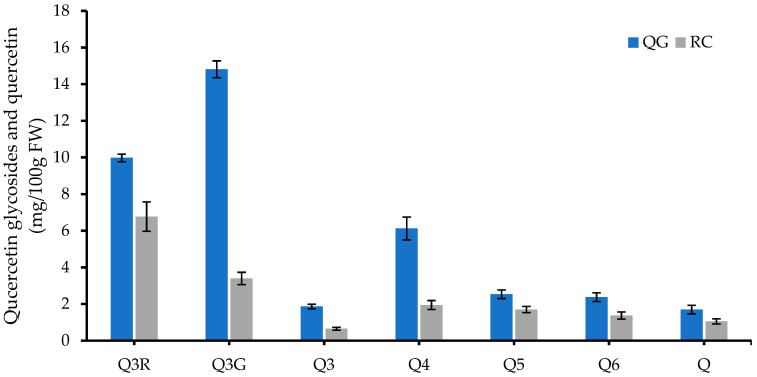
Individual quercetin glycosides and quercetin in RC and QG at day 0. Q3R, quercetin-3-rutinoside; Q3G, quercetin-3-glucoside; Q3, quercetin-3-glucosyl-xyloside; Q4, quercetin-3-xyloside; Q5, quercetin-3-rhamnoside; Q6, isorhamnetin-3-rutinoside; Q, quercetin. Data are presented as mean ± SE (*n* = 6).

Quercetin-3-rutinoside (rutin) was the main quercetin glycoside (40% of TQC) identified in RC, while quercetin-3-glucoside was the predominant quercetin glycoside (38% of TQC) in QG (Figure 5). Venter, et al. [44] reported the presence of quercetin-3-glucoside, quercetin-3-rutinoside, quercetin-3-xyloside, and quercetin-3-rhamnoside in 11 Japanese plum varieties grown in South Africa. In that study, except for one variety, quercetin-3-glucoside was found to be the main quercetin glycoside in all tested plum varieties, which is in agreement with our findings. Jang, et al. [45] also reported the presence of quercetin-3-glucoside, quercetin-3-rutinosude, quercetin-3-xyloside, quercetin-3-rhamnoside, and quercetin in Japanese plums. Furthermore, isorhamnetin-3-rutinoside and the above-mentioned quercetin glycosides could be detected in 17 European plum varieties [46]. The presence of quercetin-3-glucosyl-xyloside in Japanese plums was also reported by Suleria and co-workers [39]. However, no data have been published for quercetin glycosides in plumcots, neither content nor composition.

### 2.5. Carotenoids

**Figure 6:**
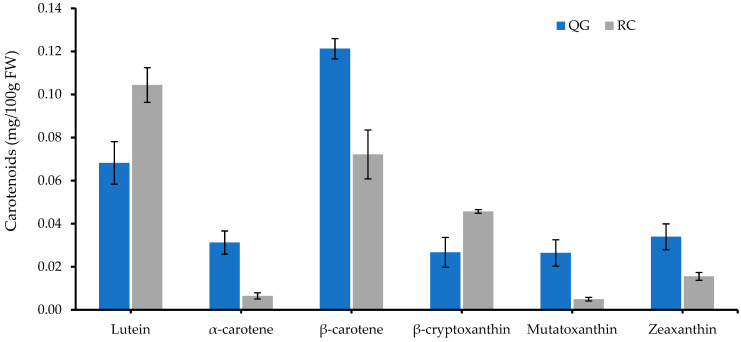
Individual carotenoids in RC and QG at day 0. Data are presented as mean ± SE (*n* = 4–6).

Carotenoid (lipophilic pigments) levels in plums are relatively low when compared with anthocyanins (hydrophilic pigments), and the colour given by the carotenoids is masked by the dark red-purple colour of the anthocyanins. However, six different carotenoids could be identified in QG and RC, with β-carotene as the predominant carotenoid in QG (39% of total carotenoids) and lutein as the predominant carotenoid in RC (42% of total carotenoids) (Figure 6).

**Figure 7:**
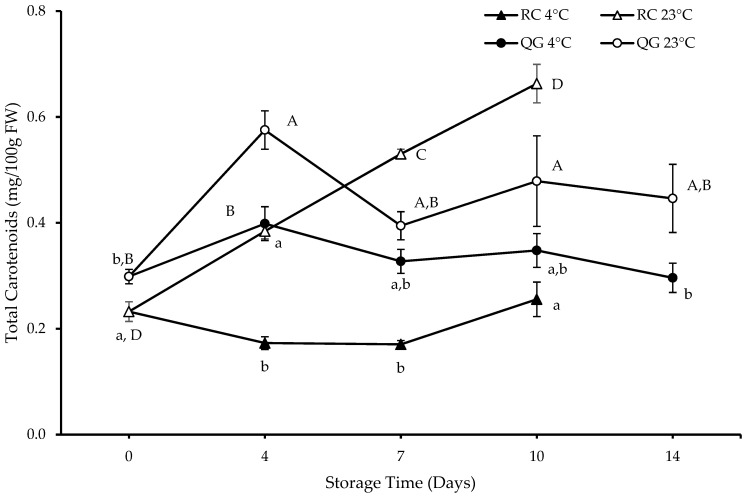
Total carotenoids (calculated as lutein equivalents) in RC and QG. Data are presented as mean ± SE (*n* = 4–6). Different uppercase and lowercase letters indicate significant (*p* < 0.05) differences between storage days for each storage temperature.

Previous QG studies have reported similar carotenoid profiles [21,35]. The initial (day 0) carotenoid concentration in QG was slightly higher than that in RC (0.30 vs. 0.23 mg/100 g FW), as shown in Figure 7. Furthermore, there was no obvious “trend” (increase or decrease) in carotenoids during storage, neither in QG nor RC, except for RC at 23 °C: a continuing increase from day 0 to day 10, from 0.23 to 0.66 mg/100 g FW. There were significant differences (*p* < 0.05) in total carotenoid concentration between the storage temperatures of 4 °C and 23 °C on all storage days for both RC and QG.

However, the carotenoid concentration in QG and RC was considerably lower than that reported for apricots (2.10–32 mg/100 g FW, [47]), which are regarded as a ‘very high’ (>2 mg/100 g) carotenoid containing fruits [48].

### 2.6. Sugars

**Figure 8:**
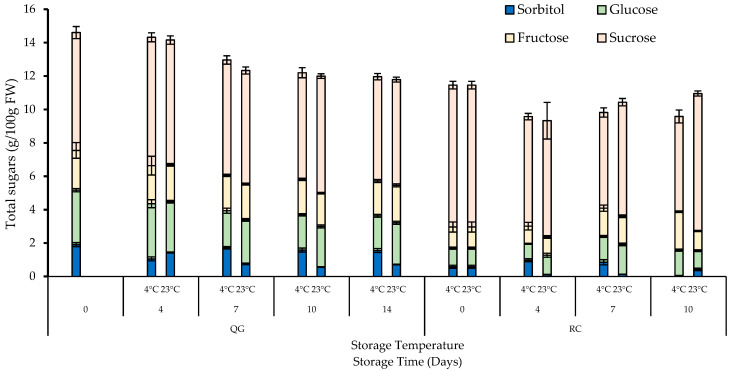
Individual sugars in RC and QG during storage. Data are presented as mean ± SE (*n* = 6).

The main sugars identified in QG and RC are sucrose, fructose, glucose, and the sugar alcohol sorbitol (Figure 8). According to the literature, the prominent sugars in plums and apricots are fructose, glucose, sucrose, and sorbitol (sugar alcohol) as well as raffinose, rhamnose, arabinose, galactose, and xylose as the minor ones [49,50]. One study has reported that ‘Harmony’ plumcot, a cross between ‘Soldam’ plum and ‘Harcot’ apricot, contained fructose, glucose, sucrose, and sorbitol [51], which is in agreement with our results. Sucrose was the main sugar in QG and RC. Sorbitol is an important sugar alcohol used in diabetes management as it has a low caloric value compared to other sugars [52]. However, the dietary relevance of sorbitol in QG and RC should be investigated further since this sugar alcohol is only a minor sugar component in both fruits.

**Figure 9:**
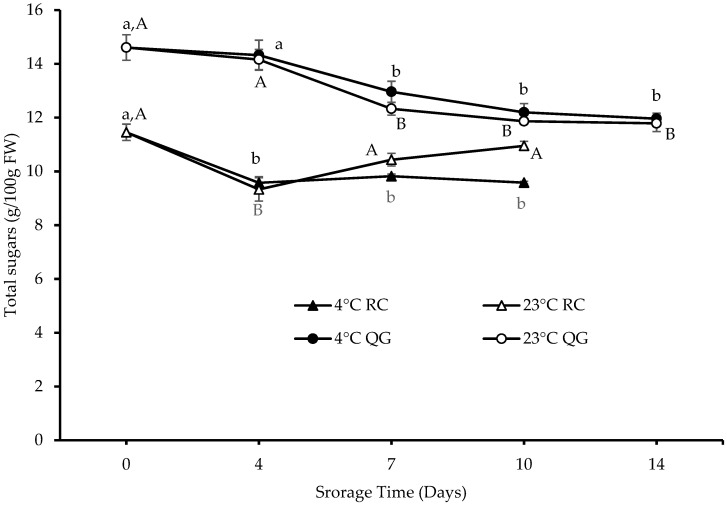
Total sugars in QG and RC during storage. Data are presented as mean ± SE (*n* = 6). Different uppercase and lowercase letters indicate significant (*p* < 0.05) differences between storage days for each storage temperature.

QG had a higher initial total sugar content than RC (15 vs. 11 g/100 g FW). Furthermore, the total sugar content decreased gradually with storage time in QG, whereas a decrease (day 4) and increase (day 7 and day 10, only at 23 °C) could be observed in RC (Figure 9). In QG, no significant difference (*p* > 0.05) was observed in total sugars between the two storage temperatures across all days. In contrast, in RC, a significant difference (*p* < 0.05) in total sugars between the two storage temperatures was only observed after 10 days of storage.

In contrast to our QG results, an overall increase in total sugars in two Japanese plum varieties stored at 0 and 5 °C and no significant changes at 20 °C were reported by Singh and co-workers [53]. However, significant variations in the sugar accumulation (metabolism) can occur during the ripening process and storage of plums, depending on their genotype, storage temperature, and maturity [53,54,55].

## 3. Materials and Methods

### 3.1. Plant Materials

RC plumcots were harvested at Applethorpe, QLD, Australia, in 2019, at the commercial maturity stage, and QG plums were purchased from a commercial grower in Cobram, VIC, Australia, during the 2019/2020 harvesting season (Figure 1). RCs were transported to the laboratory on the same day of harvest, and QG plums were stored at 2 °C for one week before being transported to the same laboratory at Coopers Plains, QLD, Australia. Fruits were sorted and randomly divided into 9 groups of 12 fruits for RC and 9 groups of 9 fruits for QG. For each fruit type, four groups of fruit were stored at 4 °C, another four groups at 23 °C, and one group was used as the reference group for day 0. QG plums were stored for 14 days, and RCs were stored for 10 days. These storage durations were determined after conducting a preliminary study on the storage stability (textural and physicochemical) of RC and QGP to ensure that each variety maintains its quality and suitability for human consumption.

After 4, 7, 10, and 14 days of storage, one group of fruit from each storage temperature and each fruit type was withdrawn randomly. For each sampling date and fruit type, the following analyses were performed immediately. Fruit weight and colour were determined individually for each fruit and 4 RC plumcots and 3 QG plums from each group were pureed separately to measure TSS and TA. The remaining fruits were freeze-dried, ground into powder (MM400 Retsch Mixer Mill, Haan, Germany), and stored at −20 °C for the analysis of phytochemicals and sugars.

### 3.2. Chemicals

Commercial standards of cyanidin-3-glucoside (C3G), Q3G, gallic acid, and (all-E)-lutein were purchased from Sapphire Bioscience Pty Ltd. (Redfern, NSW, Australia); fructose, glucose, sucrose, and sorbitol were purchased from Sigma-Aldrich (Sydney, NSW, Australia). All other chemicals and solvents (HPLC grade) were purchased from Sigma-Aldrich or Merck (Darmstadt, Germany). Deionized water (Millipore Australia Pty Ltd., Kilsyth, VIC, Australia) was used throughout the study.

### 3.3. Methods

#### 3.3.1. TSS and TA

TSS was measured in triplicate from the puree of a single fruit with a digital refractometer (PR-101, ATAGO, Tokyo, Japan). TA (as g of malic acid equivalents per 100 g of fresh sample weight) was determined in triplicates with 0.2 g of puree by an automatic titration system (Metrohm Dosimat 765, Karl Fischer, Metrohm, Herisau, Switzerland) with 0.1 N NaOH up to pH 8.2.

#### 3.3.2. Colour

The skin colour of each RC and QG was measured along the equator of the fruit at three random points. The automatic average calculated by the Konica Minolta CR-40 Chroma Meter (Konica Minolta, Osaka, Japan) was used as values of chroma (C*) and hue angle.

#### 3.3.3. Total Phenolic Concentration, Anthocyanins, and Quercetins

- Extraction

Anthocyanins and non-anthocyanin phenolics were extracted according to Hong, et al. [56], with slight modifications as previously described by Kodagoda et al. [21]. Approximately 0.2 g of freeze-dried RC and QG powders were extracted with 5 mL of cold methanol–Milli-Q water (MQ-water) (80:20, *v*/*v*) and 0.1 M HCl. Then, the mixture was shaken on a reciprocating shaker (RP1812, Paton Scientific, Victor Harbor, SA, Australia) for 10 min at 200 rpm/min, followed by centrifugation (Eppendorf Centrifuge 5804, Eppendorf, Hamburg, Germany) at 3900 rpm for 10 min at 4 °C. The supernatant was collected, and the residue was re-extracted twice using the same procedure as described above. Finally, the supernatants were combined and filtered through a 0.2 μm membrane filter before determining the total phenolic content, anthocyanins, and quercetins. All extractions were carried out in triplicate.

- TPC

TPC was determined using the Folin–Ciocalteu method [57] with modifications as previously described by Phan et al. [58]. Briefly, 25 uL of the gallic acid standard (21, 42, 63, 84, and 105 mg/L) or plant extract or MQ water were added to different wells of a 96-well plate in triplicate. Then, to each well, 100 μL of 10% Folin–Ciocalteu reagent and 100 μL of 0.7 M Na_2_CO_3_ were added. The plate was shaken for 15 s and incubated in a dark place at room temperature for 15 min, and the absorbance was read at 700 nm on a micro-plate absorbance reader (Sunrise, Tecan, Maennedorf, Switzerland). TPC was expressed as milligrams of gallic acid equivalents per 100 g of fresh sample weight (mg GAE/100 g FW) based on the external gallic acid standard curve.

- Anthocyanins and quercetins

**Figure 10:**
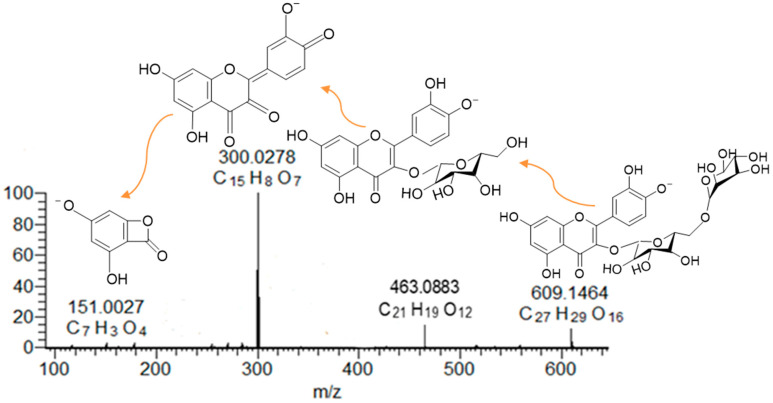
Mass spectrum and inserts of fragmentations of peonidin-3-rutinoside.

**Figure 11:**
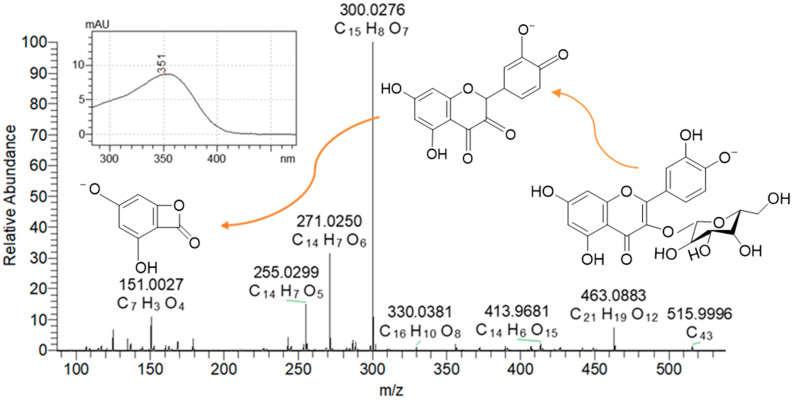
Mass spectrum and inserts of fragmentations and UV spectra of quercetin-3-glucoside.

Anthocyanins and quercetins in RC and QG were determined using a Nexera X2 UHPLC system connected to an M30A diode array detector (DAD) and an 8060 triple-quadrupole mass spectrometer (Shimadzu, Kyoto, Japan), as previously described by Hong, Netzel and O’Hare [56]. The identification of the analysed compounds was further confirmed by a Q-Exactive high-performance benchtop quadrupole-Orbitrap LC–MS/MS (Thermo Fisher Scientific, San Jose, CA, USA). The heated electrospray ionisation (HESI-II) sprayer was optimized as follows: capillary temperature, 250 °C; auxiliary gas heater temperature, 300 °C; sheath gas flow rate, 15 units; auxiliary gas unit flow rate, 5; spray voltage, 2500 V (for ESI−); and nitrogen gas consumption of 8 L/min. The Q-Exactive mass spectrometer was operated with full-scan MS and all-ion fragmentation (AIF) monitoring mode in both positive and negative resolving power of 70,000 FWHM at *m*/*z* 200. Full-scan MS was selected in a range of *m*/*z* 120−1200; the automatic gain control (AGC) was set at 3 × 10^6^ and the injection time was set to 200 ms. The scan rate was set at 2 scans/s, and the untargeted MS/MS analysis was performed at collation energies of −35 eV in positive mode and 35 eV in negative mode. Peaks were identified based on the characteristic wavelengths of the UV-visible absorption maxima, calculated exact mass, and retention times (Table 2, Figure 10 for anthocyanins, and Table 3 and Figure 11 for quercetin, respectively). MS data were analysed using the Quan/Qual Browser Xcalibur 4.0 (Thermo Fisher Scientific, USA).

Chromatographic separation was carried out on a reverse phase Acquity UPLC BEH C18 column (150 × 2.1 mm i.d., 1.7 μm particle size; Waters, Dublin, Ireland) as previously described by Hong, et al. [59], with slight modifications. Briefly, the column temperature was maintained at 50 °C, and the flow rate was set at 0.25 mL/min. The elution programme consisted of 100% mobile phase A (92.5% water, 6.5% acetonitrile (ACN), 1% formic acid (FA)) as the initial eluant for 1 min, followed by a linear gradient from 100% to 75% mobile phase A over 20 min, and 40% mobile phase A in 5 min. The column was purged for 1 min with 90% mobile phase B (ACN, 1% FA), conditioned for 1 min, and re-equilibrated for 4 min. The DAD spectrum was scanned from 200 to 800 nm and monitored at 520 and 350 nm for the identification and quantification of anthocyanins and quercetins, respectively. External calibration curves of C3G and Q3G were used to quantify anthocyanins and quercetins.

#### 3.3.4. Carotenoids

- Extraction

The extraction of carotenoids from QG and RC was adapted from the method described by Hong, et al. [60]. Approximately 0.5 g of freeze-dried QG and RC was mixed with 6 mL of ethanol containing 0.1% butylated hydroxytoluene (BHT) and vortexed for 30 s. Then, 3 mL of 5% NaCl and 10 mL of 20% dichloromethane (DCM)–80% n-hexane (extracting solvent) were added and vortexed for 10 s. The solution was centrifuged at 3900 rpm for 10 min at room temperature, and the top layer was removed. Samples were re-extracted twice using 10 mL of DCM/n-hexane, sonicated for 10 min, and centrifuged, and then the top layer was separated. All the top layers were combined and evaporated at 30 °C for 45 min using a centrifugal evaporator (DUC-23050-H00, miVac, Genevac, Ipswich, England), and the remaining solvent was evaporated until dryness by using a nitrogen stream. For the saponification, dry extracts were reconstituted with 2 mL of methanol/MTBE (1:1, *v*/*v*) with 0.1% BHT and 3 mL of 15% KOH (*w*/*v*) in methanol. Extracts were shaken for 60 min on an RP1812 Paton Scientific orbital shaker at 100 rpm at room temperature. To terminate the saponification reaction, 7 mL of 2 M phosphate buffer (pH 3) was added immediately. Then, to the final mixture, 10 mL of extracting solvent was added and centrifuged at 3900 rpm for 2 min. The top layer was removed, evaporated to dryness under a nitrogen stream, and stored at −20 °C until analysis. All extractions were performed in triplicate under dim light.

- Analysis

Carotenoids were analysed according to Hong, Takagi, and O’Hare [60] with slight modifications. Dried saponified samples were reconstituted with 2 mL of methanol/MTBE (1:1, *v*/*v*) containing 0.1% BHT and filtered through a 0.2 μm syringe filter before being transferred into HPLC vials. Quantification was carried out by a Shimadzu UHPLC system consisting of a system controller (SCL-40), three pumps (LC-40Dx3), an autosampler (SIL-40Cx3), a column heater (CTO-40C), a diode-array detector (SPD-M30A) and two degassers (DGU-405). UV/Vis spectra of the extracted carotenoids were recorded between the range of 200–800 nm, and carotenoids were analysed and processed at 450 nm using either the Shimadzu UHPLC system operated by LabSolutions Ver.5.85 (Shimadzu) or a Waters Acquity^TM^ UPLC system operated by Empower^TM^ software Ver 3.7.0 (Waters, Milford, MA, USA). Chromatographic separation was performed by a YMC C30 analytical column (250 × 4.6 mm, 3 μm particle size; Kinesis, Brisbane, QLD, Australia) connected to a YMC C30 guard column. The column temperature was maintained at 25 °C, and 5 μL of each extract was injected. The gradient programme consisted of mobile phase A (88% methanol, 10% MTBE, 2% water, 0.1% FA (*v*/*v*/*v*)) and mobile phase B (88% MTBE, 10% methanol, 2% water, 0.1% FA (*v*/*v*/*v*)). The elution was carried out at a flow rate of 0.6 mL/min for a period of 40 min with a gradient of 80% mobile phase A for 1 min, from 20% B to 25% B in 18 min, and 30% B in 9 min. This was followed by a sharp increase to 70% mobile B in 4 min and held for 2 min, conditioned for 1 min, and re-equilibrated for 5 min. Lutein and β-carotene were identified by comparing their retention times, molecular masses (Shimadzu LC-MS/MS), and specific absorption spectra with those of commercial standards. An external calibration curve of lutein was used to quantify the carotenoids as lutein equivalents.

#### 3.3.5. Sugars

Sugars in QG plum were extracted, identified, and quantified according to Hong, et al. [61], as previously reported by Kodagoda et al. [21]. For the identification and quantification of sugars in RC, freeze-dried powder (0.2 g) was extracted with 10 mL of 70% aqueous methanol (*v*/*v*). The mixture was vortexed, sonicated for 30 min at room temperature, shaken at 200 rpm/min for 10 min in a horizontal reciprocating shaker, and centrifuged at 3900 rpm for 10 min. Then, the supernatant was collected, and the pellet residue was re-extracted using the same procedure. The supernatant was combined and diluted 40 times using 50% ACN (*v*/*v*). To prepare for sugar analysis, the diluted sample was filtered through a 0.2 μm membrane filter. The sugar profiles of the RC tissues were determined using a Shimadzu Nexera X2 UHPLC system coupled with a Shimadzu MS-8045 triple-quadrupole mass spectrometer with Lab Solutions software Ver.5.85 (Shimadzu) according to Hong, Phan, and O’Hare [61]. 

#### 3.3.6. Statistical Analysis

Data were subjected to analysis of variance (ANOVA) using IBM SPSS Statistics for Windows, version 28.0 (IBM Corp., Armonk, NY, USA). Significant differences (*p* < 0.05) between means were determined using Tukey’s HSD. Data are presented as mean ± standard error (SE).

## 4. Conclusions

‘Rubycot’ has an average anthocyanin concentration compared to the high-anthocyanin ‘Queen Garnet’ plum. Both fruits contain carotenoids in relatively low amounts. Storage at ambient temperature (23 °C) enhances the nutritional value of both by significantly increasing bioactive anthocyanins and quercetins. However, further investigation into the sensory properties and consumer acceptance of the stored fruits is essential. This future research is crucial for establishing ‘Rubycot’ as a novel, storage-stable plumcot cultivar in Australia and internationally.

## Figures and Tables

**Table 1:** Changes in physiochemical properties of QG and RC stored at 4 and 23 °C.

Physicochemical Property	Storage Temperature (°C)	‘Queen Garnet’ Plum					‘Rubycot’ Plumcot			
		Storage Time (Days)								
		0	4	7	10	14	0	4	7	10
TSS	4	15.1 ± 0.03 ^a,A^	15.5 ± 0.12 ^a,A^	15.1 ± 0.09 ^a,A^	15.8 ± 0.06 ^a,A^	15.6 ± 0.52 ^a,A^	15.6 ± 0.67 ^a,A^	16.2 ± 0.95 ^a,A^	16.7 ± 1.53 ^a,A^	16.9 ± 0.55 ^a,A^
	23	15.1 ± 0.03 ^a,A^	15.3 ± 0.28 ^a,A^	16.1 ± 0.67 ^a,A^	15.8 ± 0.75 ^a,A^	15.6 ± 0.24 ^a,A^	15.6 ± 0.67 ^a,A^	16.8 ± 0.38 ^a,A^	16.2 ± 0.21 ^a,A^	15.1 ± 0.18 ^a,A^
TA	4	1.27 ± 0.00 ^a,A^	1.3 ± 0.07 ^a,A^	1.2 ± 0.11 ^a,A^	1.22 ± 0.04 ^a,A^	1.22 ± 0.03 ^a,A^	2.18 ± 0.24 ^a,A^	2.39 ± 0.04 ^a,A^	2.11 ± 0.02 ^a,A^	2.19 ± 0.13 ^a,A^
	23	1.27 ± 0.00 ^a,A^	1.09 ± 0.00 ^a,b,B^	0.96 ± 0.01 ^b,c,B^	0.85 ± 0.04 ^b,c,B^	0.72 ± 0.05 ^c,B^	2.18 ± 0.42 ^a,A^	1.94 ± 0.51 ^a,B^	1.54 ± 0.09 ^a,B^	1.67 ± 0.06 ^a,B^
Chroma	4	9.18 ± 0.46 ^a,A^	10.8 ±0.94 ^a,A^	9.58 ± 0.78 ^a,A^	10.1 ± 0.65 ^a,A^	9.57 ± 0.68 ^a,A^	23.0 ± 0.68 ^b,A^	24.1 ± 0.41 ^a,b,A^	25.4 ± 0.61 ^a,A^	23.4 ± 0.56 ^a,b,A^
	23	9.18 ± 0.46 ^a,A^	6.15 ± 1.62 ^b,B^	4.68 ± 1.54 ^b,c,B^	3.68± 0.69 ^c,B^	3.36 ± 0.13 ^c,B^	23.00 ± 0.68 ^a,A^	19.8 ± 0.74 ^a,b,B^	18.7 ± 1.41 ^b,A,B^	17.9 ± 0.28 ^b,B^
Hue	4	14.7 ± 1.21 ^a,A^	6.58 ± 1.19 ^b,B^	6.56 ± 1.00 ^b,A^	5.46 ± 1.43 ^b,A^	5.42 ± 1.48 ^b,A^	39.4 ± 2.83 ^a,A^	40.4 ± 3.28 ^a,A^	39.7 ± 2.37 ^a,A^	39.8 ± 2.04 ^a,A^
	23	14.7 ± 1.21 ^a,A^	11.9 ± 1.62 ^a,b,A^	8.20 ± 1.27 ^b,c,A^	9.12 ± 0.69 ^b,c,A^	5.82 ± 0.74 ^c,A^	39.4 ± 2.83 ^a,A^	43.5 ± 1.87 ^a,A^	38.7 ± 1.89 ^a,A^	37.8 ± 2.72 ^a,A^

Data are represented as mean ± SE (*n* = 4 for TSS and TA, and *n* = 10 for chroma and hue). For each parameter, different upper-case letters (A,B) show significant differences (*p* < 0.05, Tukey’s HSD test) between the two storage temperatures (4 and 23 °C) for each storage day; different lowercase letters (a–c) within each row show significant differences (*p* < 0.05, Tukey’s HSD test) between storage days.

**Table 2:** Identification of anthocyanins in RC and QG by LC-MS/MS and UHPLC-DAD.

Anthocyanin	Elution Time (min)	λ_max_ (nm) *	Precursor Ions (*m*/*z*) (M+)		Fragments	Molecular Formula
			Observed	Theoretical		
cyanidin-3-glucoside	5.36	515	449.1085	449.1078	399.9877 (C_21_H_4_O_9_), 287.0549 (C_15_H_11_O_6_), 193.0496 (C_10_H_9_O_4_)	C_21_H_21_O_11_^+^
cyanidin-3-rutinoside	6.46	517	595.1667	595.1657	287.0550 (C_15_H_11_O_6_)	C_27_H_31_O_15_^+^
peonidin-3-glucoside	9.06	521	463.1241	463.1235	286.0472 (C_15_H_10_O_6_)	C_22_H_23_O_11_^+^
peonidin-3-rutinoside	9.68	519	609.1821	609.1814	517.0426 (C_26_H_13_O_12_), 516.0350 (C_26_H_12_O_12_), 427.0113 (C_23_H_7_O_9_), 301.0707 (C_16_H_13_O_6_)	C_28_H_33_O_15_^+^

* UHPLC-DAD.

**Table 3:** Identification of quercetin derivatives/quercetin in RC and QG by UHPLC-MS/MS and UHPLC-DAD *.

Compound	Elution Time (min)	λmax (nm) *	Precursor Ion (*m*/*z*)		Fragments
			Observed	Theoretical	
quercetin-3-rutinoside (rutin)	10.87	354	609.1463 ^a^	609.145 ^a^	300.0276 [C_15_H_8_O_7_]^−^, 255.0299 [C_14_H_7_O_5_]^−^, 151.0027 [C_7_H_3_O_4_]^−^
			611.1615 ^b^	611.1607 ^b^	303.0500 [C_15_H_11_O_7_]^+^, 464.9974 [C_25_H_6_O_10_]^+^, 304.0530 [C_22_H_8_O_2_]^+^
quercetin-3-glucoside	11.24	351	463.0886 ^a^	463.0871 ^a^	300.0276 [C_15_H_8_O_7_]^−^, 271.0249 [C_14_H_7_O_6_]^−^, 255.0299 [C_14_H_7_O_5_]^−^, 243.0294 [C_13_H_7_O_5_]^−^, 151.0027 [C_7_H_3_O_4_]^−^
			465.1035 ^b^	465.1028 ^b^	303.0500 [C_15_H_11_O_7_]^+^, 229.0496 [C_13_H_9_O_4_]^+^, 153.0183 [C_7_H_5_O_4_]^+^
quercetin-3-glucosyl-xyloside	11.84	355	595.1303 ^a^	595.1294 ^a^	271.0249 [C_14_H_8_O_7_]^−^, 463.0884 [C_21_H_19_O_12_]^−^, 299.0201 [C_15_H_7_O_7_]^−^, 255.0299 [C_14_H_7_O_5_]^−^
			597.1460 ^b^	597.1450 ^b^	392.9906 [C_19_H_5_O_10_]^+^, 374.9799 [C_19_H_3_O_9_] ^+^, 303.0500 [C_15_H_11_O_7_]^+^
quercetin-3-xyloside	12.39	351	433.0778 ^a^	433.0765 ^a^	300.0276 [C_15_H_8_O_7_]^−^, 271.0249 [C_14_H_7_O_6_]^−^, 243.0294 [C_13_H_7_O_5_]^−^, 151.0027 [C_7_H_3_O_4_]^−^
			435.0929 ^b^	432.0922 ^b^	303.0500 [C_15_H_11_O_7_]^+^, 229.0496 [C_13_H_9_O_4_]^+^, 153.0182 [C_7_H_5_O_4_]^+^
quercetin-3- rhamnoside	13.11	359	447.0935 ^a^	447.0922 ^a^	356.9707 [C_12_H_5_O_13_]^−^, 271.0250 [C_14_H_7_O_6_]^−^, 151.0027 [C_7_H_3_O_4_]^−^
			449.1085 ^b^	449.1078 ^b^	374.9799 [C_19_H_3_O_9_]^+^, 392.9904 [C_16_H_13_O_7_]^+^, 229.0495 [C_13_H_9_O_4_]^+^, 153.0182 [C_7_H_5_O_4_]^+^
isorhamnetin-3-rutinoside	13.7	347	623.1624 ^a^	623.1607 ^a^	315.0514 [C_16_H_11_O_7_] ^-^
			625.1775 ^b^	625.1763 ^b^	392.9904 [C_19_H_5_O_10_]^+^, 317.0656 [C_16_H_13_O_7_]^+^, 229.0496 [C_13_H_9_O_4_]^+^, 153.0182 [C_7_H_5_O_4_] ^+^
quercetin	17.4	369	301.0356 ^a^	301.0343 ^a^	145.0285 [C_9_H_5_O_2_]^−^, 117.0334 [C_8_H_5_O]^−^

^a^ mass signals were detected in negative ion mode; ^b^ mass signals were detected in positive ion mode. * UHPLC-DAD.

## Data Availability

The original contributions presented in the study are included in the article, and further inquiries can be directed to the corresponding author.
